# A Scoping Review of the Evidence on Health Promotion Interventions for Reducing Waterpipe Smoking: Implications for Practice

**DOI:** 10.3389/fpubh.2018.00308

**Published:** 2018-11-05

**Authors:** Karen Gardner, Rachael Kearns, Lisa Woodland, Mariela Silveira, Myna Hua, Milena Katz, Klara Takas, Julie McDonald

**Affiliations:** ^1^Public Service Research Group, University of New South Wales Sydney, NSW, Australia; ^2^Centre for Primary Health Care and Equity, University of New South Wales, Sydney, NSW, Australia; ^3^South Eastern Sydney Local Health District, New South Wales Department of Health, Sydney, NSW, Australia

**Keywords:** waterpipe, smoking, health promotion, health intervention, tobacco control

## Abstract

**Background:** Waterpipe tobacco smoking is a traditional method of tobacco use, especially in the Eastern Mediterranean Region (EMR), but its prevalence is growing worldwide, especially among young people. Although often perceived as less harmful than other methods of tobacco use because the smoke passes through water, accumulating evidence shows harmful effects and that some smokers become addicted. Interventions that deglamourise and denormalise use have been recommended but little is known about the range and impact of different health prevention and promotion interventions.

**Methods:** A scoping review of literature was undertaken to explore the breadth of literature and assess the range and impact of community based health promotion interventions for waterpipe smoking. Searches were conducted in Medline, Embase, CINAHL, Psychinfo, and the Cochrane database of systematic reviews. Interventions were classified using a health promotion framework and data extracted on the aspects of prevention/promotion addressed; key strategies employed, evidence of effectiveness or impact on behavior change as well as barriers to implementation and perceived success factors.

**Results:** Ten studies were included in the review. They include brief interventions to increase quit rates; community campaigns to raise awareness and increase knowledge; web based health education and skill development to increase perceived risks and intention to quit; as well as studies that evaluated product labeling and opportunities for policy interventions to create healthy environments.

**Conclusions:** The evidence base is small but growing. Brief interventions for waterpipe users, community campaigns, and web based tailored information can modify perceptions of addiction and increase intentions to quit. Product labeling may be an effective policy tool to curb waterpipe smoking. A range of policy interventions have been identified but not evaluated.

## Background

Waterpipe smoking is a traditional method of tobacco use, practiced originally in the Middle East but becoming increasingly popular worldwide, particularly among young people and women (1, 2). Recent estimates of the rapid increase in waterpipe use across the world suggest a very high prevalence among school and university students in Middle Eastern countries, as well as among groups of Middle Eastern descent in western countries including the USA and Australia (3). Although often perceived as less harmful than other types of tobacco use because the smoke passes through water, there is growing evidence of health risks and harm associated with toxicity, associations with lung cancer, periodontal disease and other conditions, as well as the development of dependence in some users (2–4).

Systematic reviews of interventions addressing waterpipe use have largely focused on behavioral and treatment interventions with very limited attention to health promotion and prevention (5, 6). Despite growing consensus that waterpipe smoking is a public health issue and calls from the World Health Organization for action to intervene (7) little is known about how best to deliver health promotion interventions that can increase awareness of potential harm and improve community capacity for action. A recent review of interventions for waterpipe smoking cessation (5) concluded that evidence-based information about waterpipes' addictive and harmful properties should be developed and disseminated in order to deglamourize and denormalize its use. Ward et al. (8) have also argued that programs need to address the unique features of waterpipe smoking e.g., its cultural significance, social uses, and intermittent use pattern as well as the characteristics and motivations of users who want to quit.

We conducted a scoping review to identify and describe the range of literature on health promotion initiatives and to assess evidence of impact and the key mechanisms, barriers and enablers to implementation. Specific questions were:

- What types of community based health promotion interventions for waterpipe smoking have been trialed?
- What aspects of prevention do they seek to address and what mechanisms for change are employed?
- Which of these have been shown to be effective, for which population groups/communities and in which contexts?

## Methods

The review follows the scoping methodology outlined by Arksey and O'Malley (9). Consistent with this methodology, the review was conducted in 5 steps. Step 1 involved developing the research questions; Step 2 identifying relevant studies; Step 3 selecting studies; Step 4 charting data; and Step 5 collating, summarizing and reporting results. A project team consisting of health promotion practitioners, policy makers and researchers was established to develop the research question and oversee the study.

### Search strategy

A comprehensive search strategy and set of search terms is contained in Supplementary File 1. Search terms included “waterpipe” or “narghile” or “arghile” or “shisha” or “goza” or “narkeela” or “hookah” or “hubble bubble” AND a combination of “health promotion” or “health intervention” or “health education” or “social marketing” or “health knowledge” (see Supplementary File 1). Searches were conducted in April 2016 in Medline, Embase, CINAHL, Psychinfo and the Cochrane database of systematic reviews. Hand searching of key journals and citations from key papers was also conducted.

### Inclusion criteria for study selection

Studies of health promotion interventions, using the WHO health promotion definition (10) (http://www.who.int/topics/health_promotion/en/) were included. Studies that focused on treatment interventions, attitudes and prevalence, studies not in English, and gray literature were excluded.

### Charting data

Two authors (RK, KG) extracted data. Details of the study population and context, aims and methods, intervention strategies, mechanisms if available, key success factors and barriers to implementation, and impacts were recorded. Studies were classified across the spectrum of health promotion (11) see the Northern Territory Health Promotion Framework) from medical approaches such as brief intervention that focus on individuals, through behavioral approaches that aim to improve knowledge and skills, to socio-environmental activities that focus on creating healthy communities, settings, and environments. Formal quality assessment was not conducted, consistent with the scoping review methodology and because of the small numbers of papers and heterogeneity of topics and study types.

## Results

**Figure 1:**
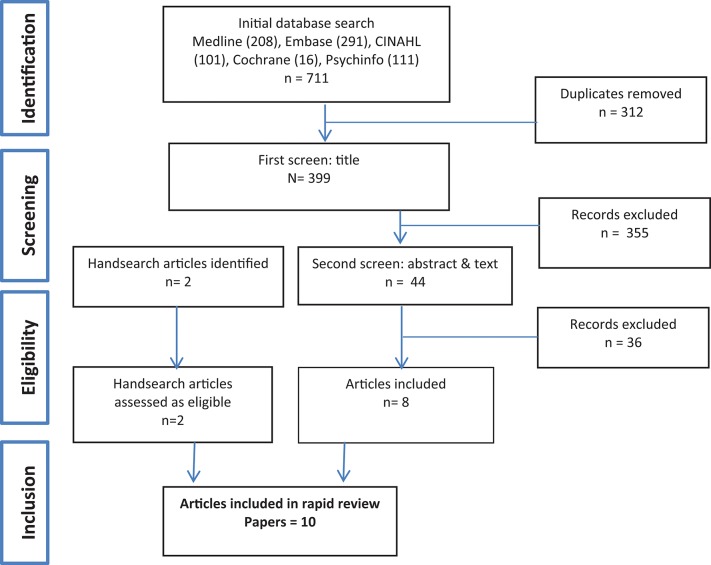
Search strategy results.

The initial search identified 711 references, of which 312 duplicates were removed, leaving 399 papers that were subject to first screen title review (Figure 1). A further 388 papers that did not meet our criteria were excluded. Many of these were papers reporting on various aspects of prevalence; health knowledge, perceptions, and behaviors; treatments and policies related to waterpipe control. Ten studies were included in the final review.

As shown in Table 1, included articles were: a study of a brief behavioral intervention to increase quit rates (12); three studies examining web based health education and skill development initiatives to increase cessation as well as perceived risks and intention to quit (13–15); a community education campaign to raise awareness and increase knowledge (16); as well as five studies that evaluated product labeling or implementation of tobacco control legislation as policy opportunities to create healthy environments (17–21).

**Table 1:** Intervention type.

**Screening Indiv Risk assessment and immunization**	**Health information and social marketing**	**Health education and skill development (online education)**	**Community awareness and action (community campaign)**	**Settings and supportive environment (policy)**
Brief Intervention – brief (1 in-person session and 3 phone calls) or intensive (3 in-person sessions and 5 phone calls) behavioral cessation treatment delivered by a trained physician in a clinical setting (12)	Nil	Web-based program that provides tailored feedback to increase smoking knowledge and reduce cigarette and nargila smoking behaviors among Arab college/university students in Israel (13). Effects of web-based information on worry as precursor to quit (14). Reduction of cigarette smoking via iQUIT: A web-based program using podcasting and text messaging in adolescents (15).	Community intervention for tobacco and sheesha to increase knowledge of risk (16)	Potential policy interventions (17). Evaluation of health warning labeling of waterpipe products and accessories in 9 countries (18). Text-only and pictorial health warning labels and their location on different parts of waterpipe smoking devices (19). Existing tobacco legislation should be amended to accommodate WTS, including consideration of licensing the industry (20). The majority of tobacco control policies have exemptions for waterpipe in the US (21).

### Intervention characteristics

Table 2 describes the characteristics of studies. As shown, Asfar‘s (12) randomized controlled trial tested the feasibility and potential efficacy of brief interventions for waterpipe smoking cessation for willing-to-quit adults who had smoked waterpipe >3 times per week in the last year (but not cigarettes), comparing brief and more intensive interventions delivered by a trained physician in a clinical setting in Syria. Both the brief and more intensive interventions resulted in prolonged abstinence and brief interventions were as effective as more intensive interventions for willing to quit waterpipe users.

**Table 2:** Study details.

**References**	**Study population and context**	**Study description/ Aim Method**	**Intervention type and strategies Mechanisms for change if discussed**	**Key success factors and barriers to implementation (Lessons learned)**	**Measured impacts and outcomes**	**Conclusions**
Asfar et al. (12)	Study population: Adult waterpipe smokers (*n* = 50) who smoked waterpipe > 3 times per week in the last year, did not smoke cigarettes, and were interested in quitting. Context: An outpatient cessation clinic, located in a private general hospital in central Aleppo, Syria.	Aim: To develop and pilot a behavioral intervention for willing-to-quit waterpipe users to: (1) evaluate the feasibility of the intervention (2) test its potential efficacy (3) determine the adequacy of intervention “dose” in terms of contact frequency. Methods: A pilot, two arm, parallel group, randomized, open label trial. Participants were randomized to receive either brief or intensive behavioral cessation treatment. Primary end point was abstinence at 3 months assessed by self-report and exhaled carbon monoxide levels of < 0.10 ppm. Secondary end points were 7 day point-prevalent abstinence and adherence to treatment. Participants completed a semi-structured process evaluation interview.	Brief intervention: Education/counseling sessions by a trained physician and follow up phone calls Brief (1 in-person 45 min session and 3 phone calls) participants educated about health effects and consequences of waterpipe use, encouraged to set a quit date, taught basic stimulus control and contingency management strategies to quit and prevent relapse. or Intensive (3 in-person 45 min sessions and 5 phone calls) behavioral cessation treatment delivered by a trained physician in a clinical setting. The same approach as the brief arm, but provided enhanced counseling in using a problem-solving approach. This included instruction and practice in anticipating high-risk situations, a relapse prevention plan, and using cognitive and behavioral coping strategies, self-rewards, and social support. Both groups: Written educational self-help materials.	The strongest predictor of cessation at the 3-month follow-up was having made a successful quit attempt for at least 1 month during the last year. Could indicate participants developed quitting skills and/or enhanced their self-efficacy that were useful during the current quit attempt. The most helpful strategies: - encouraging physical activity (71.4%) - receiving educational information (71.4%) - rules of relapse prevention (57.6%) - getting social support (47.6%). Suggestions for improvement were more frequent, longer contacts, using medication. Almost half of participants were interested in receiving a group smoking cessation intervention. Participants were interested in receiving more phone calls than in-person sessions.	30% of participants were fully adherent to treatment which did not vary by treatment group. Prolonged abstinence in the brief and intensive interventions at 3-months were 30.4 and 44.4%, respectively. Previous success in quitting (*OR* = 3.57; 95% CI = 1.03–12.43) predicted cessation. Higher baseline readiness to quit, more confidence in quitting, and being unemployed predicted a better adherence to treatment (all *p*-values 0.05). The first session in future trials should be provided immediately after randomization to capitalize on smokers' high (but soon-to-dwindle) motivation.	Brief behavioral cessation treatment for waterpipe users appears to be feasible and effective. Cessation rates were not significantly different in the intensive and brief treatment arms. A single in-person session of education and advice from a trained professional, along with brief telephone follow-up, may be as effective as a more intensive intervention for willing-to-quit waterpipe users.
Essa-Hadad et al. (13)	Study population: Arab college/university students aged 18 years of age or older (*n* = 225; mean age 25; more than 2/3 female) Context: Israel.	Aim: To examine the acceptability and feasibility of a pilot web-based program using tailored feedback to increase smoking knowledge and reduce cigarette and nargila smoking behaviors. Methods: A mixed-methods study using both quantitative (pre/post-test study design) and qualitative tools. A post-test at 1 month following participation in the intervention. Primary outcomes: Self-reporting of cigarette and nargila smoking behavior. Increases in cigarette and nargila smoking knowledge. Focus group sessions assessed acceptability and preferences related to the web-based program. Secondary outcome: intention to quit, reason for wanting to quit, and seeking of professional help to quit.	Health education and skill development: A pilot web-based program providing tailored feedback. Consists of (1) a self-administered online questionnaire on cigarette and nargila smoking behavior and knowledge (2) tailored health education material delivered via text and videos.	Participants preferred tailored feedback. Compared with non-tailored messages, tailored health messages are more likely to be read and remembered, saved and discussed with others, perceived as interesting and personally relevant, and designed especially for the recipient. Primary reason given for trying to quit smoking was to improve health status. The majority (50/56, 89%) of participants, reported preference of the computer program over other traditional means of health education. Participants reported the feedback to be relevant, effective, clear and to the point, and interesting. The majority (49/56, 88%) of participants reported that the feedback regarding nargila smoking was most useful and interesting. Participants agreed there is very little awareness and knowledge regarding nargila. smoking. The majority (40/56, 71%) agreed that nargila smoking was socially and culturally acceptable.	225 participants-response rate of 63.2% (225/356)-completed the intervention at baseline and at 1-month post-study. Statistically significant reductions in nargila smoking (*P* = 0.001) were found but not for cigarette smoking. The tailored intervention reduced nargila smoking from 58.2% at baseline to 22.2% at the 1-month follow-up. It also resulted in statistically significant increases in the intention to quit cigarette smoking (*P* = 0.021). No statistically significant increases in knowledge were seen at 1-month post study.	A tailored web-based program may be a promising tool to reduce nargila smoking among Arab university students in Israel. The tailored web intervention was not successful at significantly reducing cigarette smoking or increasing knowledge. However, the intervention did increase participants' intention to quit smoking. Participants considered the Web-based tool to be an interesting, feasible, and highly acceptable strategy.
Lipkus et al. (14)	Study population: College students, aged 18 years or older (mean age 18), who had smoked waterpipe at least once during the last month. Majority Caucasian men. Study 1 (*n* = 70) Study 2 (*n* = 110) Context: 6 college and university campuses in central North Carolina.	Aim: To modify perceived risks and worry about waterpipe tobacco smoking. Methods: Two web-based studies providing college waterpipe users with information on (1) spread of and use of flavored tobacco in waterpipe and (2) harms of waterpipe smoking. Study 1 (*N* = 91) tested the “incremental” effects on perceptions of risk and worry. Study 2 (*N* = 112) tested the effects on perceptions of risk and worry of reviewing information about harms of waterpipe smoking compared to a no information control group. Outcomes: Between group differences in perceived and factual knowledge of harms and addictive potential of waterpipe use, perceived risk of physical harm and of becoming addicted, and desire to quit. Effects of intervention on self-reported use at 6 months. In Study 1 only the percentage of participants who reported no longer using waterpipe assessed.	Health education: Online Study 1: Experimental group: viewed 20 PowerPoint slides on smoking waterpipe and harms. Control group: shown 8 slides (information on harms excluded). Study 2: Experimental group: viewed 15 slides. Excluded information discussing the spread and popularity of waterpipe and the use of flavored additive in tobacco. Control group: no information. Mechanism: Enhancing accurate knowledge to increase perceived risk and worry about waterpipe tobacco smoking.	Across studies and conditions, participants viewed the information as understandable (mean scores of 5.65–5.95), credible (4.75–5.76), and personally relevant (4.20–5.56). The receipt of harm information produced significant change in each mediator (Perceived risk/Perceived worry of harm and addiction). A change in each mediator produced change in desire to quit, controlling for treatment. The direct effect of treatment (harm information) no longer produced changes in desire to quit when controlling for each mediator separately, suggesting complete mediation.	Pooling data from both studies, participants who received information about the harms of waterpipe smoking (Study 1 only) reported statistically significant greater perceived risk and worry about harm and addiction and expressed a stronger desire to quit. In Study 1, 62% of participants in the experimental group versus 33% in the control group reported having stopped waterpipe use. The experimental condition from Study 1 may be most effective to promote cessation in weekly and monthly users.	These are the first studies to show that perceptions of addiction and harm from waterpipe use can be modified using minimally intensive interventions; such interventions show promise at decreasing waterpipe use.
Pearlstein and Friedman (15)	Study population: 40 adolescent smokers aged 18–24 who were ready to quit. 79% of participants reported using a hookah or water pipe to smoke tobacco in addition to cigarettes. Context: An adolescent ambulatory health centre and internet.	Aim: To evaluate an internet delivered smoking cessation program. Methods: Self-selected enrolment from health centre clients via word of mouth, health centre website, advertising, local health care providers, and the iQUITwebsite. Outcomes: Self report reduction in number of cigarettes per day, reduction in the number of days per month smoking, and reduction in client CO levels.	Health education: Online motivational based smoking cessation counseling delivered by a Nurse Practitioner, certified as a Tobacco Dependence Treatment Specialist using podcasting and text messaging. Key topics on the podcasts were: setting a quit date, avoiding triggers, managing cravings, nicotine replacement, managing stress, and relapse prevention. Daily text messages were offered as additional support for the first 30 days during the program.	Unclear which technology was more helpful, podcasting versus text messaging. Further investigation is needed to determine if this technology could help reduce smoking among young people only using waterpipe.	At commencement, no participants smoked 0 cigarettes per day (CPD); 32% reported 6–10 CPD; 27% reported 11–20 CPD; and 7.5% reported smoking >20 CPD. At 1 month 11% reported 0 CPD; 44% reported 2-5 CPD, 22% reported 6–10 CPD, none reported 11–20 CPD, and 5% reported more than 20 CPD. Carbon monoxide readings still in progress. Six-month follow-up surveys still in progress.	Smoking cessation delivered to adolescents using web-based technology, podcasts, and text messaging support led to a modest reduction in the number of cigarettes used per day and the number of total days of cigarette use per month.
Mohlman (16)	Study population: Six villages of between 10,000 and 20,000 people that had at least one primary, prep and secondary school, a health clinic and a mosque. Context: Egypt.	Aim: To improve knowledge of the hazards of smoking and environmental tobacco smoke and to change attitudes and behaviors at the community and household level. Methods: Randomized controlled trial. Villages that met criteria randomly selected. Interviewer facilitated survey results from before and after the intervention period were analyzed in pair wise comparisons with data from control villages.	Community awareness and action (community campaign): Materials on smoking and passive smoking hazards and training of local people to deliver a multi-prong approach: (1) Primary school students participated in activities aimed at preventing initiation of smoking. (2) Preparatory and secondary school students engaged in an experiential learning program to develop social skills to handle peer pressure to smoke. (3) Engaged mosques and churches in educating their communities about smoking hazards and ETS and in raising smoking as a sinful behavior. (4) Female social change agents provided information to adult women in the home on the negative health effects of tobacco use and ETS. They taught them how to better protect themselves and their children from ETS through a standardized message sensitive to cultural family dynamics.	The intervention group showed greater increase in understanding dangers of smoking cigarettes and waterpipe and became more proactive by limiting exposure to smoke and enacting bans at home. The most significant increase in response to the question why quit among both the intervention and control was cited as children's health.	The intervention increased knowledge of harm; did not lead to a decrease in smokers but modified where smokers smoked and increased non-smokers advocacy for the own and their families' health.	Community interventions that seek to reduce environmental exposure through smoking bans, education and empowering people to ask smokers to stop are effective.
Morris et al. (17)	Study population: Policies related to waterpipe smoking Context: United States	Aim: To identify potential policy interventions to reduce youth hookah use.	Settings and supportive environment: - Increasing price/tax - Health warnings via labels on tobacco products and advertisements. - Extend regulation of flavored tobacco to hookah. - Smoke free environment laws - Restricting internet and mail-order access.	Studies of youth and young adults have found that predictors of smoking hookah are the same as those for cigarettes, including social acceptability, having friends and family members who smoke, and perceiving that smoking a waterpipe is not harmful. Established interventions to reduce youth cigarette smoking should be effective for reducing waterpipe smoking.		Tobacco flavor regulation: Would likely make hookah less appealing, particularly to youth. Smoke free laws: Decreases the perception of smoking as an acceptable behavior, promotes cessation and discourages youth initiation. The presence of hookah lounges creates and reinforces a community norm accepting of waterpipe smoking. Internet purchases: Expanded restrictions on credit processing for Internet purchases and shipping tobacco products would make waterpipe less accessible to youth.
Nakkash and Khalil (18)	Study population: All waterpipe tobacco products, waterpipe accessories. Context: Lebanon and a sample from Dubai (United Arab Emirates), Palestine, Syria, Jordan, Bahrain, Canada, Germany, and South Africa.	Aim: To evaluate current health warning labeling practices on waterpipe tobacco products and related accessories. Methods: Observation study examining health warning messages on waterpipe products.	Settings and supportive environment: Product health warning labeling.		The majority of products from Lebanon had textual health warning labels covering on average only 3.5% of total surface area of the package. Misleading descriptors were commonplace on waterpipe tobacco packages and related accessories.	There are no WHO FCTC compliant waterpipe-specific health warning labels on waterpipe tobacco products and related accessories. Introducing health warnings on waterpipe tobacco products and accessories will probably have worldwide public health benefits.
Islam et al. (19)	Study population: Adult waterpipe smokers (*N* = 367). Context: Large United States university.	Aim: To test the effectiveness of various text-only and pictorial health warning labels and their location on waterpipe devices. Methods: An internet-based survey.	Settings and supportive environment: Health warning labeling.		Text-only messages and pictorial labels warning about harm to children were the most effective in motivating waterpipe smokers to think about quitting. In terms of warning label location, the base, mouthpiece and stem are all equally noticeable locations.	Placing waterpipe-specific labels on waterpipe devices may be an effective policy tool to curb waterpipe smoking.
Primack et al. (21)	Study population: Municipal, county, and state level tobacco control policies Context: 100 largest cities in the United States.	Aim: To assess whether waterpipe smoking is affected by smoke free laws introduced in the 100 most populous cities in the US in 2011 or whether these laws may have intentionally or unintentionally exempted waterpipe. Methods: Analysis of municipal, county, and state law applying to the 100 largest US cities. A summary policy variable on how current tobacco control policies might apply to HTS was developed and used in a multinomial logistic regression to determine associations between community-level sociodemographic variables and a policy outcome variable.	Settings and supportive environment: Smoke free environments.	Although 3/4 of the largest US cities disallow cigarette smoking in bars, nearly 90% may permit HTS via exemptions.	73 cities had comprehensive anti-tobacco legislation in place on the municipal, county or state level that disallowed cigarette smoking in freestanding bars. However, 69 of these cities may allow HTS via exemption. Only 4 cities had clean air laws with no exemption for HTS.	Closing the gap in clean air regulation may significantly reduce exposure to waterpipe smoking.
Jawad (20)	Study population: Municipal, county, and state level tobacco control policies Context: London, United Kingdon.	Aim: To explore industry characteristics, experiences with enforcement and tobacco legislation compliance in London, UK. Methods: In-depth telephone interviews with 26 local authority (LA) staff from 14 London boroughs.	Settings and supportive environment: Enforcement and tobacco legislation compliance.	Successful methods for enforcing legislation included a synchronized, multiagency approach; however, this was inconsistently implemented across boroughs. Many LA staff believe licensing waterpipe premises would improve surveillance and control the industry's proliferation. Most waterpipe premises were generally noncompliant with most aspects of tobacco legislation, mainly due to disproportionately low fines and unclear legislation enforcement guidance.	The waterpipe industry is unregulated in many London LAs, mainly due to lack of resources. These problems may also occur in other large cities worldwide.	Existing tobacco legislation should be amended to accommodate waterpipe smoking including consideration of licensing the industry. More research is needed to gain a full understanding of the waterpipe tobacco industry and its impact on other global cities.

Three web based educational interventions targetting adolescents and university students in Israel and the United States who smoked cigarettes and waterpipe also showed some success. Essa-Hadad et al. (13) found that a web-based program providing tailored feedback to increase smoking knowledge and reduce cigarette and waterpipe smoking behaviors among Arab college/university students in Israel was highly acceptable to participants. The program significantly reduced waterpipe smoking from 58.2% at baseline to 22.2% at the 1-month follow-up, and while it did not result in a reduction to cigarette smoking, or increase knowledge, it was found to influence participants' intention to quit cigarettes. Lipkus (14) studied an online education intervention among American college students who had smoked waterpipe at least once in the last month. It successfully modified perceived risks, increased worry about waterpipe smoking and resulted in a reported reduction in waterpipe smoking among some participants. Pearlstein (15) found that an online motivational based smoking cessation program “iQUIT”delivered over a 6 month period to adolescents who reported smoking both cigarettes and waterpipe, using web-based technology, podcasts, and text messaging support, yielded a modest reduction in the number of cigarettes used per day and the number of total days of cigarette use per month. Reductions in waterpipe use were not reported.

A randomized controlled trial of a multi-pronged community intervention in six Egyptian villages by Mohlman (16) delivered education and training to women, students, and religious leaders to improve knowledge of harm related to smoking and second hand smoke and to change behavior and attitudes to waterpipe. While the intervention did not lead to a decrease in smoking (tobacco or waterpipe), it did increase knowledge of harm, modified where smokers smoked and increased non-smokers advocacy for their own and their families' health.

The remaining five studies addressed aspects of tobacco control policies for creating supportive environments to curb waterpipe use. Morris (17) identified a number of regulatory and policy levers that may result in making waterpipe smoking less appealing and available to young people. Among these were regulating tobacco flavorings to enforce removal of sweetners and additives; implementing smoke free laws to decrease the perception of smoking as acceptable, promote cessation, discourage initiation and prevent reinforcement of a community norm; and expanding restrictions on credit processing for internet purchases and shipping tobacco products to make them less accessible to youth. Nakash (18) evaluated health warning labeling practices on waterpipe tobacco products in Lebanon, Dubai, Palestine, Syria, Jordan, Bahrain, Canada, Germany, and South Africa. A lack of appropriate health warning labels on waterpipe tobacco products and accessories, misleading descriptors, and misreporting of tar and nicotine labels were identified. Islam (19) surveyed students in a large university in the United States to test the effectiveness of text-only and pictorial health warning labels and their location on waterpipe devices. Warnings about harm to children were found to be the most effective in motivating waterpipe smokers to think about quitting. The base, mouthpiece and stem were seen by participants as equally noticeable locations. Primack (21) evaluated municipal, county, and state level smoke free laws introduced in the 100 most populous cities in the United States in 2011 to assess whether waterpipe smoking is included or been intentionally or unintentionally exempted. Sixty-nine of Seventy Three cities were found to allow HTS via exemption. Jawad (20) explored industry characteristics, experiences with enforcement and tobacco legislation compliance in London through in-depth telephone interviews with 26 local authority (LA) staff from 14 boroughs. He found low levels of compliance with all forms of regulation due to disproportionately low fines and unclear legislation enforcement guidance.

### Study populations

Interventions targeted different population groups. The brief intervention specifically targeted ready-to-quit adult waterpipe smokers who did not smoke cigarettes. Web based educational programs targeted students and young people who smoked waterpipe as well as cigarettes. The community intervention targeted whole communities in six Muslim villages in Pakistan but specifically young people, religious leaders and women as the change agents. Policy and legislative changes deliver messages and create safe environments for whole populations. The studies reported here were conducted in the United States, UK, Lebanon, Dubai (United Arab Emirates), Palestine, Syria, Jordan, Bahrain, Canada, Germany, and South Africa.

### Outcomes

Motivational and behavioral outcomes were assessed in relation to: increased periods of abstinence in adults (12); increasing worry and intention to quit waterpipe among university students (14); and reductions in waterpipe smoking among young people (13, 14). In the community study, Mohlman (16) found increases in knowledge, advocacy and protective behaviors but no reductions to waterpipe smoking behaviors. Studies of product labeling or tobacco control legislation were not linked to behavioral outcomes so their impacts remain unknown.

### Mechanisms for change

Only one study specifically assessed the impact of strategies on a mechanism for change. Lipkus (14) found that the receipt of harm information produced significant change in perceived risk as well as perceived worry of harm and addiction, and each was associated with changes in desire to quit. Thus the authors conclude that strategies targeting cognitive and emotional responses to harm and addiction can be modified and that these emotional changes underlie intentions which are proximal to behavioral changes.

## Discussion

The evidence base relating to the impact of health promotion programs on individual and community change is very small. The majority of studies (5/10) focused on the policy and legislative environment and few of these measured impact on outcomes related to smoking. Only one brief intervention was identified, one community level intervention and three health education programs targeting young people. There is a gap in studies trialing health information and social marketing strategies. The only reference to work in this area identified in our search was a major campaign launched in Turkey in 2014 which included television and radio advertisements, outdoor materials, brochures, handouts, newspaper inserts, internet and social media strategies, but although an evaluation was planned it had not yet been reported (TMPD 2014).

Overall, this review indicates that behavioral health promotion interventions have been successful in raising awareness of smoking harm and increasing concern and worry as a precursor to quit in both youth (14) and community populations (16) and directly influencing waterpipe smoking behavior (12, 13). Unfortunately Pearlstein (13) did not report waterpipe smoking outcomes separately from cigarettes so it is unclear whether waterpipe practices were affected.

Both adults and young people are important target audiences. Young people are the fastest growing users of waterpipe (1) and since American data suggest that most adults become addicted during adolescence (17) increasing intention to quit among young people is important. As a practice that is often done at home in some communities, influencing parents to take action to protect children is also an important vehicle for change. The community level study reported here (16) sought to influence mothers' behavior to limit family exposure to smoke, with some success. Further work might explore potential for influencing the behavior of fathers as parents and role models.

Web based information strategies appear to be an appropriate way of providing information to young people. In the studies reviewed here, young people reported preference for these over traditional education methods and valued tailored information and feedback which they perceived as interesting and personally relevant. Health messages were more likely to be read and remembered, saved and discussed with others when tailored to their specific interests. Improving health status was a relevant message for young people since the primary reason given for trying to quit smoking was to improve their health (13).

Few studies reported on the mechanisms they sought to influence, but it is clear that each of the interventions aimed to provide participants with personal resources to bring about change in some aspect of the rules or reasoning through which people understood, undertook and experienced smoking waterpipe. According to Lacouture (22) a mechanism is *hidden but real, is an element of reasoning and reactions of agents in regard to the resources available in a given context to bring about changes through the implementation of an intervention, and evolves within an open space-time and social system of relationships*. The combination of strategies employed as part of the brief interventions for adults, examined by Asfar (12), sought to reframe people's understanding of harm (I might become addicted, get sick, or die if I continue to smoke waterpipe), induce an emotional response (some level of concern or fear as a precursor to quitting) and instill a cognitive response to controlling emotions associated with quitting (I can make a plan and stick to it even when things feel tough).

Similarly educational interventions for young people sought to enhance their understanding of harm by tailoring information to their questions and to shift the sense of glamor associated with smoking that has been reported in a number of studies (5, 23). Interventions aimed to increase doubt, induce worry and subvert perceptions that smoking means “fitting in” and “being cool.” This might enable adolescents to resist peer pressure to take up smoking and find other less harmful ways of fitting in. The community intervention used a combination of these mechanisms and in addition sought to engage religious leaders to provide authority and endorse the message for people who might feel connected to smoking as a cultural practice (smoking is not necessarily an expression of my culture). The intervention also sought to engage women's sense of responsibility in protecting children by encouraging them to intervene in practices that expose their children and family members to smoke.

Most anti-tobacco legislation in the countries included in the studies reviewed in this paper allowed waterpipe smoking venues via exemption and that compliance was poor with most forms of regulation due to legal uncertainties and low levels of fines applied. Learning from international experience and exploring potential for work at the municipal, regional or state levels is warranted. Smoke free laws may be an important step in shifting community norms. Further investigation of the suitability of labeling on products is another area in which studies indicate potential for impacting young people's knowledge of harm. Price levers may also be used to decrease affordability, especially among young people who are significantly more price sensitive than adults. While this review showed that evidence of impact of policy and regulatory interventions is limited and previous studies have highlighted the multiple challenges involved in waterpipe control (24), recent policy guidance from the World Health Organization indicates a range of policy interventions and suggested actions for regulators to control waterpipe use in signatory countries (7). These extend from the types of interventions identified in studies included in this review (product labeling, price levers, smoke free laws, taxation) to include prohibitions on manufacturers from making health claims related to sheesha products; training for health and other workers; adaptation of existing advertising to address the specific context of internet based vending; modification of waterpipe products to minimize harms; and the extension of existing tobacco surveillance and monitoring systems to include waterpipe. There is evidence that some countries are beginning to call attention to addressing loopholes in legislation, including countries in the Middle East and some states in Australia.

Multipronged approaches addressing multiple mechanisms seem well placed to address the specific practices of waterpipe smoking which differ from cigarette smoking in that they are associated with sociability and relaxation rather than stress, are perceived as less harmful than other forms of tobacco use, may be seen as part of cultural practices, and for some groups start at home.

There are a number of potential limitations to this review. While we conducted a comprehensive search using key databases and hand searching, it is possible that some papers may have been missed. In addition only papers in English were included which means there could be other relevant papers. Gray literature was beyond the scope of this review and it may be that there is evidence of program impacts in evaluation and other technical reports, not available here.

## Conclusion

Brief interventions for waterpipe users, community campaigns and web based tailored information can modify perceptions of addiction and harm, increase worry and intention to quit. Product labeling may be an effective policy tool to curb waterpipe smoking. A range of policy interventions have been identified but not evaluated. There is great scope to trial many of these interventions in different contexts. Attention to mechanisms for change including those related to gender/family and social roles, culture, age, and emotions could aid implementation. Co-design of interventions with people from specific target groups may enhance the appropriateness of nuanced messages and therefore increase acceptability and the likely uptake of interventions.

## Author contributions

KG, RK, LW, MH, MS, MK, KT, and JM designed the study. KG and RK designed the review methods, ran the black literature searches and conducted the analyses. LW and MH identified further literature. KG and RK extracted data. KG drafted the manuscript. All authors read and were involved in critically revising the manuscript and all authors have approved the final manuscript.

### Conflict of interest statement

The authors declare that the research was conducted in the absence of any commercial or financial relationships that could be construed as a potential conflict of interest.
