# Supplementary material for: A Scoping Review of the Evidence on Health Promotion Interventions for Reducing Waterpipe Smoking: Implications for Practice
Source: Front Public Health. 2018 Nov 5;6:308. doi: 10.3389/fpubh.2018.00308 (PMC6230676; doi:10.3389/fpubh.2018.00308)
Supplement: Supplementary file 1 [file Table_1.DOCX]

**Supplementary File 1:**  Search strategy

**Medline**

(waterpipe or narghile or arghile or shisha or goza or narkeela or hookah or hubble bubble).mp.

AND

exp Health Promotion or intervention.mp. or exp policy/ or exp smoke-free policy/ or policy.mp. or prevention.mp. or exp Secondary Prevention/ or exp Primary Prevention/ or social marketing.mp. or exp Social Marketing/ or health education.mp. or exp Health Education/ or exp Health Knowledge, Attitudes, Practice/ or community awareness.mp.

220 references, 24 duplicates, 12 removed = 208 total

**Cinahl**

waterpipe OR narghile OR arghile OR shisha OR goza OR narkeela OR hookah OR hubble bubble

AND

health promotion OR intervention OR policy OR smoke free policy OR prevention OR social marketing OR health education OR health knowledge, attitudes, practice OR community awareness OR primary prevention OR secondary prevention

101 references, 0 duplicates = 101 total

**Embase**

(waterpipe or narghile or arghile or shisha or goza or narkeela or hookah or hubble bubble).mp.

AND

exp Health Promotion or intervention.mp. or exp policy/ or exp smoke-free policy/ or policy.mp. or prevention.mp. or exp Secondary Prevention/ or exp Primary Prevention/ or social marketing.mp. or exp Social Marketing/ or health education.mp. or exp Health Education/ or exp Health Knowledge, Attitudes, Practice/ or community awareness.mp.

306 references, 30 duplicates, 15 removed = 291 total

**Psycinfo**

(waterpipe or narghile or arghile or shisha or goza or narkeela or hookah or hubble bubble).mp.

AND

exp Health Promotion or intervention.mp. or exp policy/ or exp smoke-free policy/ or policy.mp. or prevention.mp. or exp Secondary Prevention/ or exp Primary Prevention/ or social marketing.mp. or exp Social Marketing/ or health education.mp. or exp Health Education/ or exp Health Knowledge, Attitudes, Practice/ or community awareness.mp.

111 references, 2 duplicates, 0 removed = 111 total.

**Combined database – Intervention search**

711 references, 462 duplicates, 312 removed (Endnote and manual review) = 399 total
